# Mid-infrared plasmonic multispectral filters

**DOI:** 10.1038/s41598-018-29177-0

**Published:** 2018-07-26

**Authors:** Ang Wang, Yaping Dan

**Affiliations:** 1 State Key Laboratory of Advanced Optical Communication Systems and Networks, University of Michigan-Shanghai Jiao Tong University Joint Institute, Shanghai Jiao Tong University, Shanghai, 200240 China; 2 Key Laboratory of Infrared Imaging Materials and Detectors, Shanghai Institute of Technical Physics, Chinese Academy of Sciences, Shanghai, 200083 China

## Abstract

A miniaturized mid-infrared spectral analyzer will find a wide range of applications as a portable device in non-invasive disease diagnosis, environmental monitoring, food safety and others. In this work, we report an integrated spectral analyzer that can be constructed by using Au subwavelength hole arrays as multispectral filters. The hole arrays were fabricated with CMOS compatible processes. The transmission peak of the subwavelength hole arrays is continuously tuned from 3 μm to 14 μm by linearly increasing the periodicity of the holes in each array. Fourier transform infrared (FTIR) microscopy was applied to spatially map out the transmission of the hole arrays. The results show that each hole array can selectively allow for transmission at a specific wavelength. We further constructed an IR spectral analyzer model based on the microhole multispectral filters to retrieve IR spectral information of two test samples. Our experimental results show that the spectra from the integrated spectral analyzer follow nearly the same pattern of the FTIR spectra of the test samples, proving the potential of the miniaturized spectral analyzer for chemical analysis.

## Introduction

Most chemicals have distinct absorption signatures in mid-infrared spectral range. Spectral analysis provides a highly sensitive and selective method for chemical detection, which may find a wide range of applications in gas sensing^1^, noninvasive disease diagnosis^2,3^, security monitoring^4^ and others^5–7^. For example, two-channel spectral analyzers (InfraTec) for gas sensing have been commercialized by using two separate traditional Fabry-Perot spectral filters. The filtering spectrum of Fabry-Perot filters is determined by the cavity thickness. Each cavity thickness requires one step of microfabrication. It is extremely challenging to integrate a large number of Fabry-Perot filters on chip for constructing a smart multispectral analyzer. More importantly, the “stop band” of Fabry-Perot filters is only a few micrometers wide^8^ due to the small contrast in refractive index of the available materials that are transparent in mid-infrared. It is difficult to avoid the cross-talk of the spectral filters in the wide mid-infrared spectrum range (3 µm–30 µm).

Surface plasmon resonances (SPR) of noble and transition metals are well known for their capability to enhance light intensity in visible and near infrared range^9,10^. Nanostructured metal films have been widely used to construct RGB color filters in visible spectrum^11,12^, aiming to replace the classical organic color filters in digital cameras. Analogically, metallic microhole arrays (MHAs) were also presented for enhancing transmission of mid-infrared light^13,14^, and even terahertz radiation^15,16^. Transmission peaks at specific wavelengths could be achieved by properly designing metal MHAs, providing a new approach to realize multispectral infrared photodetectors^17^. Here we report plasmonic multispectral filters in mid-infrared that are constructed using a subwavelength hole array in evaporated Au thin film. Nanohole arrays in Au thin film were previously reported as color filters in visible spectrum^18^. The plasmonic resonance in the metal film can be shifted from visible to mid-infrared range (3 µm to 14 µm) simply by increasing the size of nanoholes to microscale. The experimental results show that the microhole arrays on germanium (Ge) substrate have a main spectral peak of 60% transmittance with a full width at half maximum (FWHM) of ~1.5 µm in addition to some side spectral lobes. The transmission spatial distribution of 30 microhole arrays was also mapped out by a microscopic FTIR spectrometer. Each microhole array shows distinct spectral transmission although some crosstalk is observed for microhole arrays with close physical dimensions.

## Simulation and Experiments

In 1998, Ebbeson *et al*. first investigated a periodic subwavelength hole array in a silver film and observed an extraordinary optical transmission (EOT) phenomenon such as enhanced transmission of light through the holes and wavelength filtering due to the excitation of SPR [10]. In case of normal incidence, the peak positions, *λ*_*max*_, of the transmission spectrum of a subwavelength hole array in a hexagonal lattice are approximately given by

1 $${\lambda }_{max}=\frac{P}{\sqrt{\frac{4}{3}({i}^{2}+ij+{j}^{2})}}\sqrt{\frac{{\varepsilon }_{m}{\varepsilon }_{d}}{{\varepsilon }_{m}+{\varepsilon }_{d}}}$$

where *P* is the period of the array, *i* and *j* are the scattering orders of the array, *ε*_*m*_ and *ε*_*d*_ are the dielectric constants of the metal and the dielectric material in contact with the metal, respectively^19^.

Following the above equation, multicolor filters in visible spectrum based on nanohole arrays has been extensively investigated in the past decade^20–23^. To design a multispectral filter in mid-infrared range, finite difference time domain (FDTD) simulations were performed on an 80 nm thick Au film with periodic micro-sized apertures. The Au film is on a mid-infrared transparent Ge substrate. Since the plasmonic resonances exist at the top and bottom surface of the Au film, a thin Ge film is also covered on top of the Au film to ensure that the plasmonic resonances at both surfaces are at the same wavelength. A light source with wavelength from 2 μm to 15 μm is set polarized in x direction (see SI Fig. S1 for other polarizations) and launched perpendicularly from the thin film top. Periodic MHAs in the Au film are simulated by setting periodic boundary conditions for a unit cell.

**Figure 1:**
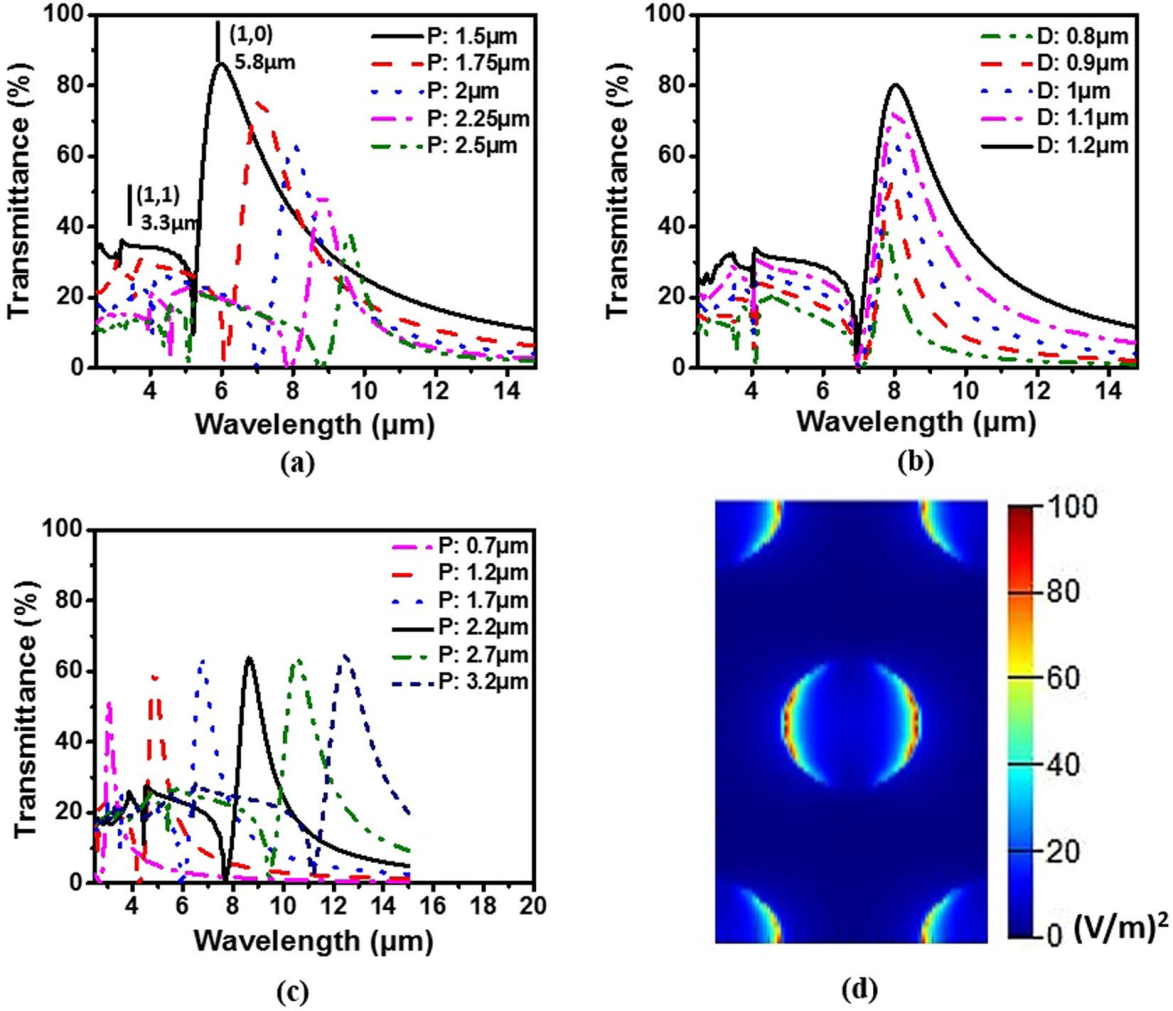
(**a**) Simulations as periodicity changes from 1.5 μm to 2.5 μm at a fixed diameter of 1 μm. (**b**) Simulations as diameter changes from 0.8 μm to 1.2 μm at a fixed periodicity of 2 μm. (**c**) Simulation results of Au MHAs with a fixed ratio 0.5 for aperture diameter to period. (**d**) Top view of the simulated E-field distribution inside the holes at peak position.

According to Eq. (1), the resonance wavelength is mainly determined by periodicity. We first set diameter at a fixed value of 1 µm and shifted the period from 1.5 μm to 2.5 μm at a step of 0.25 µm. It was found that each transmission spectrum has a main peak along with several small side lobes at shorter wavelengths. The main peak is the first order of scattering with (i = 1, j = 0) or (i = 0, j = 1), and the small side lobes are higher orders of scattering. For example, for the period of 1.5 μm, the first order is at ~5.8 μm and the second order (i = 1, j = 1) is at ~3.3 μm ($5.8/\sqrt{3}$). As the period increases, the main peak position shifts from ~5.8 μm to ~9.8 μm but the peak amplitude shows a trend of decline [Fig. 1(a)]. This is not surprising since the relative aperture area becomes smaller for a larger period (the aperture diameter is fixed). As a result, a smaller portion of light will pass through at a given incident light intensity. To investigate the effect of diameter on transmission spectrum, we performed another set of simulations by fixing the period at 2 μm and changing the diameter from 0.8 μm to 1.2 μm at a step of 100 nm. The results are shown in Fig. 1(b), from which we find that the main peak position remains nearly constant, except that the peak amplitude becomes stronger due to the fact that a larger opening at a fixed period will allow more light to transmit.

As a multispectral filter for spectral analysis, it is desirable to have a larger transmittance and a narrower FWHM to achieve a higher signal-to-noise ratio (SNR) and spectral resolution. However, as shown in Fig. 1(a,b), a higher transmittance always comes with a wider FWHM. To balance the transmittance and FWHM, we chose a ratio of 0.5 for the aperture diameter to period (see more discussions in SI Section 2). Figure 1(c) shows the simulation results based on this ratio, at which the transmittance maximum is approximately 60% and the FWHM is about 1 µm. As the period increases (diameter also increases accordingly), the main position linearly shifts from 3.5 µm to 13.5 µm and the main peak amplitude slightly increases from 50% to ~65%. For the following experimental work, we fixed the ratio of aperture diameter to period at this value. Figure 1(d) shows that the electric field intensity of the light is mainly localized near the metal edge of the aperture as the light transmits through the aperture due to metal surface plasmon polariton. This is consistent with the previously observed EOT phenomenon at visible spectral range.

**Figure 2:**
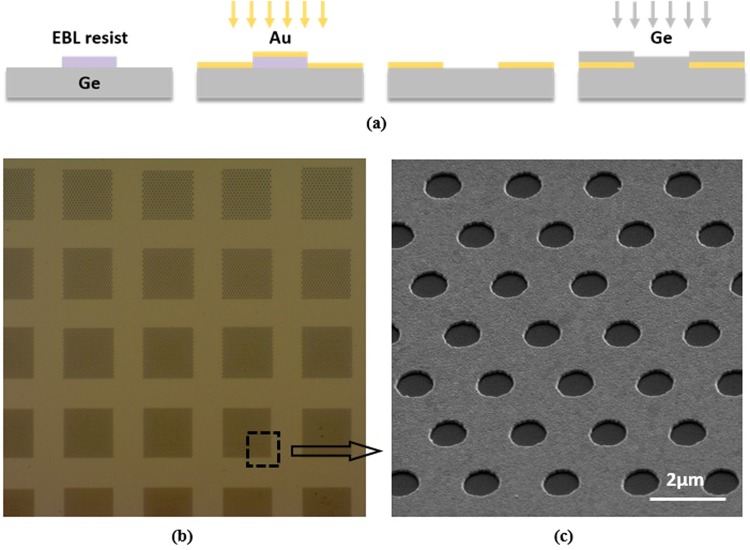
(**a**) Fabrication process of Au microhole arrays. Step I: applying EBL to write patterns in ZEP520 on Ge. Step II: depositing an 80 nm-thick Au film via electron beam evaporation. Step III: using acetone to pattern Au film. Step IV: sputter a 160 nm-thick Ge layer on the top of metal. (**b**) Overview micrograph of Au microhole arrays. (**c**) A close-up SEM image of the fabricated Au microhole array on Ge wafer.

To fabricate the multispectral filters according to the simulations above, the electron beam resist ZEP520 was first spin-cast onto the sample and patterned by electron-beam lithography (EBL, Vistec EBPG-5200) at a dosage of 155 μC/cm^2^. As shown in Fig. 2(a), we then evaporated an 80-nm thick Au film by an electron beam evaporator (Denton) on Ge substrate (1 cm × 1 cm). After that, we immersed the sample in acetone for hours, followed by a short time ultrasonic cleaning to strip the resist together with the gold film on top of it. As the final step, a 160-nm thick Ge protection layer was sputtered on the metal structure using a magnetic control sputtering system (Denton). The created apertures in the metal film are arranged hexagonally to form an array of 50 μm × 50 μm, shown in the scanning electron microscopic (SEM) image in Fig. 2(c). In total, we fabricated 30 microhole arrays with the aperture period tuning from 0.5 μm to 3.4 μm at a step of 100 nm, which are partially shown in Fig. 2(b). The circular aperture diameter is changed accordingly to ensure a constant ratio (1:2) of diameter to period.

**Figure 3:**
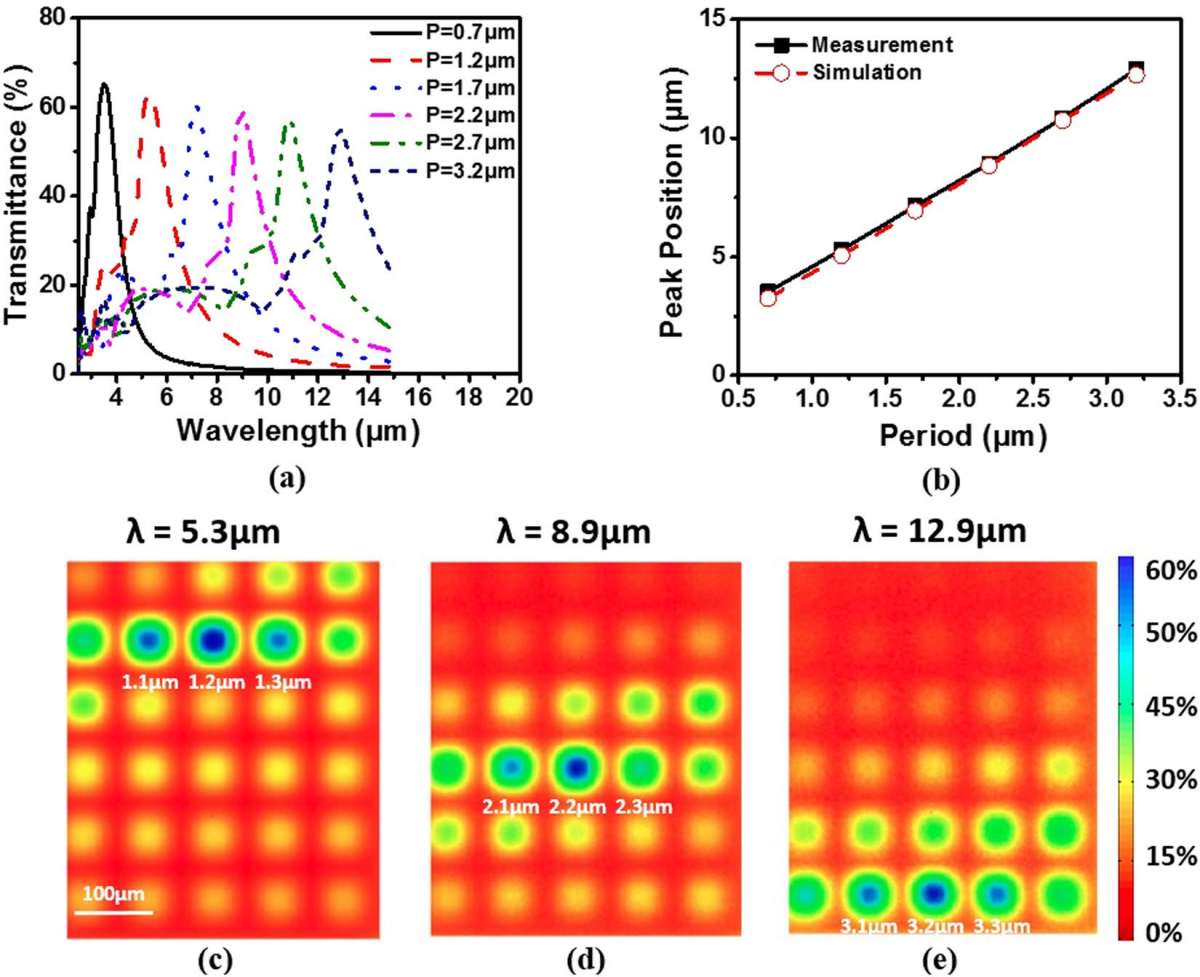
(**a**) Experimentally measured optical transmission spectra of six (out of 30 in total) microhole arrays. (**b**) Transmittance peak position as a function of the aperture period in simulation and experimental results. Mid-infrared transmission 2D maps of the MHAs are plotted at three different wavelengths: 5.3 μm (**c**), 8.9 μm (**d**) and 12.9 μm (**e**).

The transmission spectra of the fabricated Au MHAs on a Ge substrate (“sample spectra”) were measured with a microscopic FTIR spectrometer (Thermo Scientific Nicolet iN10). To remove the effects caused by atmospheric conditions and substrate, the transmission spectra of a Ge substrate (“substrate spectra”) was also recorded. The transmission spectra of the Au MHAs were obtained by dividing the sample spectra with the substrate spectra. We plot six of the total 30 spectra in Fig. 3(a). The peak amplitudes show a trend of decline as the period increases. It is probably because the small aperture size is made slightly larger than designed due to the proximity effect of electron beam exposure. The transmittance peak position as a function of the aperture period is shown in Fig. 3(b). As the period increases from 0.7 μm to 3.2 μm (in 500 nm incremental), the transmittance peak shifts from 3.5 to 13 μm, following almost exactly the same dependence with the simulations. To visualize the wavelength selectivity of the MHA filters, we image the spatial transmittance distribution of the Au film at specific wavelengths (Fig. 3(c,d,e)). The transmittance is coded in color and the image has a spatial resolution of 2 μm × 2 μm. At a wavelength of 5.3 μm, the microhole array with a period of 1.2 μm has the maximum transmittance of 60%, which is consistent with the spectrum (red dash line) in Fig. 3(b). All the other arrays show a transmittance lower than ~40% except for the adjacent two arrays. Similar patterns can be also found in the other two images in Fig. 3(d,e).

**Figure 4:**
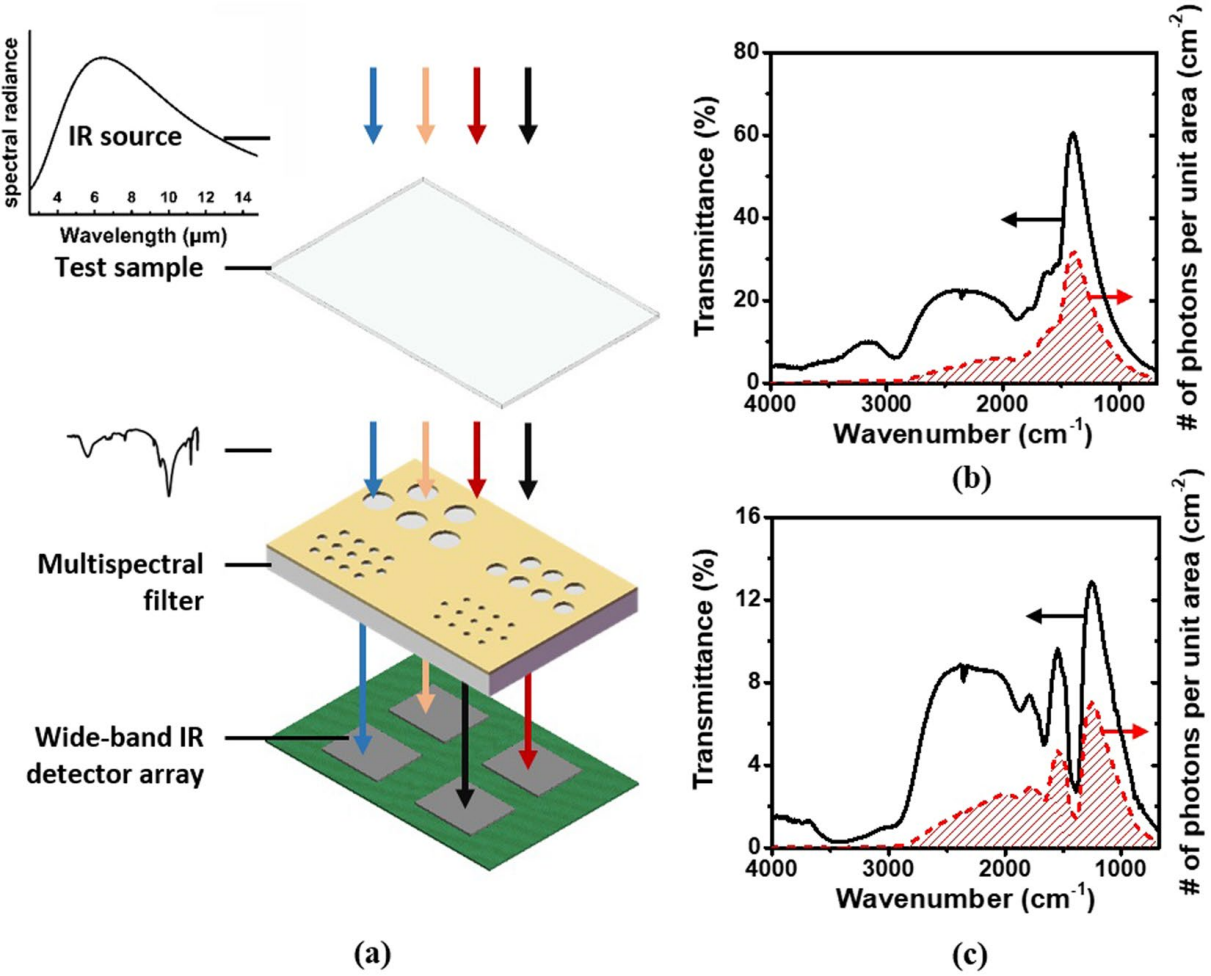
(**a**) IR spectrometric analyzer model using microhole array multispectral filters. Transmission spectrum of a microhole array (P = 1.7 μm) (**b**) without and (**c**) with a test sample, respectively.

If each microhole array is equipped underneath with a broadband mid-infrared photodetector, a chip-size spectral analyzer can be realized with appropriate signal processing technology, as shown in Fig. 4(a). In such a system, we can assume a blackbody IR light source that has a spectral radiance density governed by eq. (2).

2 $${B}_{T}(\lambda )=\frac{2{\rm{hc}}}{{\lambda }^{3}}\frac{1}{\exp (hc/{\rm{\kappa }}T\lambda )-1}$$

where h is the Plank constant, c is the light speed, κ is the Boltzmann constant, T is the absolute temperate and *λ* is the wavelength. *T *= 450 k is used here so that the maximum radiation intensity is at *λ*_*max*_ = 6.4 μm (Fig. 4(a)). Each array of microholes has a different filtering spectrum as shown in Fig. 3(a). The spectral profile for the *i*th array is represented by *F*_*i*_(*λ*). All the photodetectors have the same wavelength-dependent quantum efficiency *η*(*λ*). The transmission spectrum *B*_*T*_(*λ*)*F*_*i*_(*λ*) after the light source is filtered by a microhole array (P = 1.7 μm) is shown as the solid black line in Fig. 4(b). The transmission spectrum can be converted into the photon number per unit area, shown as the red slash profile in Fig. 4(b). Let us suppose that the sample under test has a transmission spectrum *X*(*λ*) which is the known. The transmission spectrum *B*_*T*_(*λ*)*F*_*i*_(*λ*)*X*(*λ*) after filtered by the same microhole array is shown as the solid black line in Fig. 4(c). Similarly, the transmission spectrum can be converted into the photon number per unit area, shown as the red slash profile in Fig. 4(c).

The photon number will be collected as photocurrent by photodetectors. For simplicity, we assume that the internal quantum efficiency of the photodetectors is 100%, meaning that all the photons at different wavelengths will be collected by the photodetector and converted to a photocurrent. For the case when the sample under test does not present in the optical path, the photocurrent *I*_*i*0_ for the *i*th photodetector will be expressed as Eq. (3) in which *hc*/*λ* is the photon energy.

3 $${I}_{i0}={\int }_{{\lambda }_{1}}^{{\lambda }_{2}}\eta (\lambda )\frac{{B}_{T}(\lambda ){F}_{i}(\lambda )}{{\rm{hc}}/\lambda }{A}_{c}{\rm{d}}\lambda $$

where *A*_*c*_ is the area of each microhole array filter. For the case when the sample is in the optical path, the photocurrent *I*_*i*_ for the *i*th photodetector will be written as

4 $${I}_{i}={\int }_{{\lambda }_{1}}^{{\lambda }_{2}}\eta (\lambda )\frac{{B}_{T}(\lambda ){F}_{i}(\lambda )}{{\rm{hc}}/\lambda }X(\lambda ){A}_{c}{\rm{d}}\lambda $$

Clearly, the photocurrent ratio ${R}_{i}=\frac{{I}_{i}}{{I}_{i0}}$ for the *i*th photodetector will be equal to the transmittance X(*λ*_*i*_) at the wavelength *λ*_*i*_ when the multispectral filter has an ideal performance, that is, *F*_*i*_(*λ*) = *δ*(*λ*_*i*_). However, as the spectral profiles of our microhole arrays are not delta functions (Fig. 3(a)), the spectral information of a sample cannot be accurately given by the photocurrent ratios.

For this reason, we employ the following algorithm to reconstruct the spectral information of the sample. Eq. (4) is the photocurrent for the ith photodetector after integrating all wavelengths from 3 um to 14 um (2000 points). For all photodetectors, the photocurrent vector can be written as the following equation when the test sample is not present in the optical path and the quantum efficiency is a unity at all wavelengths.

5 $${I}_{0}=FB,{I}_{0}\in {{\mathbb{R}}}^{30\times 2000}$$

where $F\in {{\mathbb{R}}}^{30\times 2000}$ is the filtering matrix for all 30 filters at the 2000 wavelengths. *F* is experimentally measured. $B\in {{\mathbb{R}}}^{2000\times 1}$ is the vector of the light source spectrum and the *i*th element in the vector is equal to ${B}_{T}({\lambda }_{i})\ast {\rm{\Delta }}\lambda /({\rm{hc}}/{\lambda }_{i})$.

Similarly, when the test sample is present in the optical path, the photocurrent vector can be written as

6 $$I=FXB,I\in {{\mathbb{R}}}^{30\times 1}$$

where $X\in {{\mathbb{R}}}^{2000\times 2000}$ is a diagonal matrix and the (*i*, *i*) element on the diagonal is the sample spectral information at the *i* th wavelength. The filtering spectral vector *FX* is experimental measured when the sample is in the optical path.

**Figure 5:**
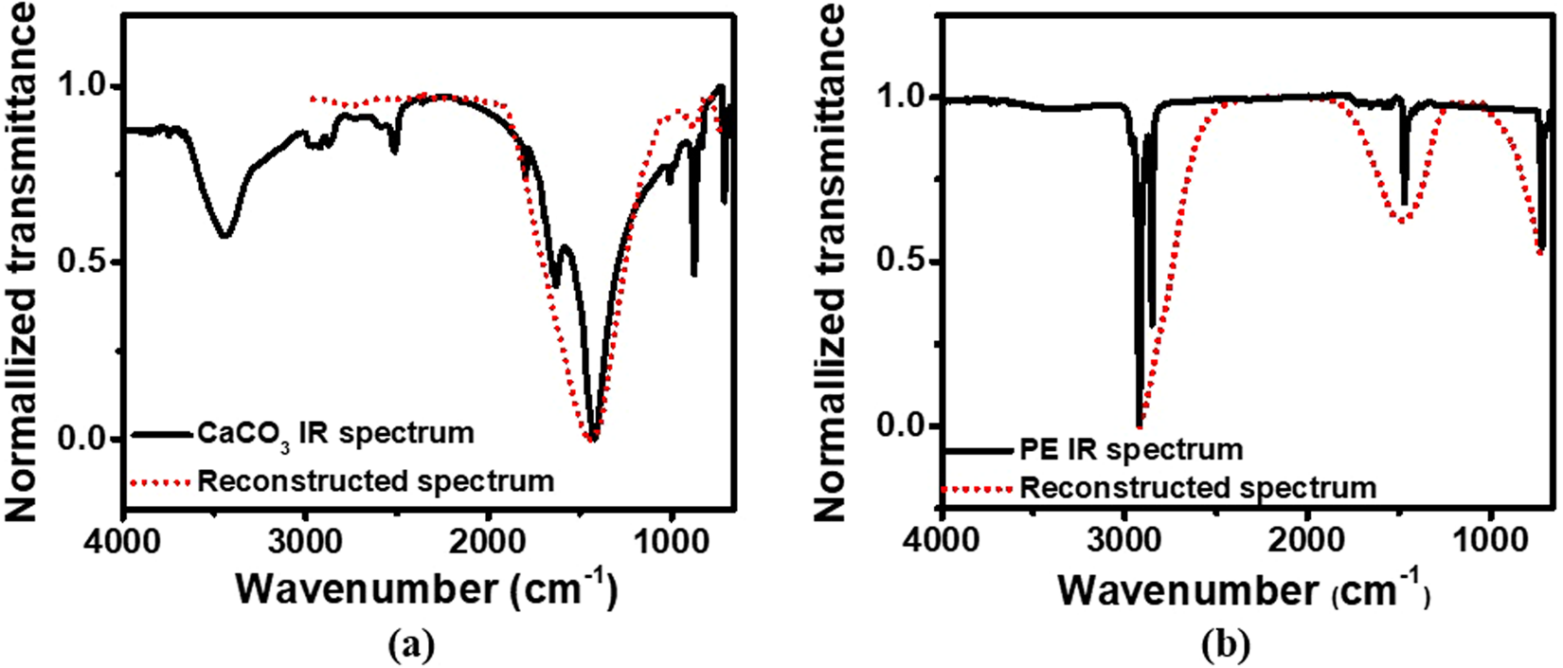
(**a**) FTIR spectrum of CaCO_3_ (solid) and reconstructed spectrum (dotted); (**b**) FTIR spectrum of polyethylene (solid) and reconstructed spectrum (dotted).

However, the matrix *X* cannot be directly solved from Eq. (6) because *F* and *B* are not reversible. Instead of directly solving Eq. (6), we can reformulate Eq. (6) into an optimization problem by finding *X* to minimize the difference between the two sides of the equation, *i.e*. $$min\{I-FXB\}$$ which is equivalent to $$min\,\{I-\,FXB{\}}^{2}=$$$${\rm{\min }}\,\{{(I-FXB)}^{{\rm{T}}}(I-FXB)\}$$. Minimizing the difference optimized the reconstructed spectrum of the sample under test. Figure 5(a,b) show the FTIR (solid) and reconstructed (dotted) spectrum for CaCO_3_ and polyethylene (PE), respectively. (We use these two chemicals as examples simply because it is easy to prepare samples since they are solid.) Clearly, our multispectral analyzer can capture the main spectral feature of both materials. Due to the relatively wide FWHM of the filtering spectrum, however, some small side spectral dips are not resolved by the spectral analyzer. These dips are buried in the main valley, resulting in an asymmetric shape of the main valley.

We should admit that such an IR spectrometric analyzer based on MHAs cannot compete with the Fourier Transform Infrared Spectroscopy (FTIR) in terms of spectral resolution. The measured filtering spectra for the MHA filters has a FWHM of ~1.5 μm on average. This limits the spectral resolution to 0.75 μm (half of the FWHM), meaning that our spectral analyzer will not be able to resolve a spectral feature narrower than 0.75 μm in FWHM (similar to the light not able to resolve an object smaller than half of the wavelength), no matter how many MHA filters are used. But the number of MHA filters, although the more the better, shall not be smaller than a lower limit. Otherwise, the integrated spectral analyzer cannot reach its full capability. To find this lower limit, we can regard the MHA filters as probes to sample the target spectral information. According to the sampling theory, the sampling rate should be at least twice the maximum bandwidth (BW) of the target information, that is,

7 $${\rm{\Delta }}k\le \frac{1}{2BW}$$

where Δk is the sampling interval in wavenumber domain. The bandwidth (BW) of the target IR transmission spectrum can be found^24^ as

8 $$BW\approx \frac{0.35}{{t}_{\tau }}$$

where *t*_*τ*_ is the change of wavenumber as the IR spectrum varies from 10% to 90% of the spectral feature (absorption peak or transmission valley). From Eqs (7) and (8), we find

9 $${\rm{\Delta }}k\le \frac{{t}_{\tau }}{0.7}$$

Let us take the IR spectrum of CaCO_3_ in Fig. 5(a) as an example where ${t}_{\tau }\approx 100\,c{m}^{-1}$, yielding a maximal sampling interval of 142 cm^−1^. In the spectral range from 4000 to 400 cm^−1^, the minimal number of filters is 26. In our case, 30 MHA filters are used, which is large enough to allow our spectral analyzer to reach its spectral resolution, although we designed the MHA filters to sample the target spectrum uniformly in wavelength.

Indeed, our integrated spectral analyzer cannot replace the FTIR spectral analyzer for the general purpose of spectral analysis. But it may be quite useful for some specific applications where the miniaturization of the device is highly desired while the spectral features are not complicated.

## Conclusion

In this work, Au MHAs were successfully fabricated on Ge substrate using electron beam lithography and evaporation. The transmission spectra of the microhole arrays were measured with a microscopic FTIR spectrometer. Spatial mappings at fixed wavelengths were applied to visualize the wavelength selectivity of the Au MHA filters. An IR spectral analyzer model based on these filters was constructed to demonstrate the feasibility of retrieving the IR spectra of test materials. The successful development of such an integrated spectral analyzer may find a wide range of applications in non-invasive disease diagnosis, environmental monitoring, food safety and others.

### Data availability

The data that support the findings of this study are available from the authors on reasonable request, see author contributions for specific data sets.

## Electronic supplementary material

supplementary information
