# Supplementary material for: Mid-infrared plasmonic multispectral filters
Source: Sci Rep. 2018 Jul 26;8:11257. doi: 10.1038/s41598-018-29177-0 (PMC6062579; doi:10.1038/s41598-018-29177-0)
Supplement: Supplementary file 1 — supplementary information [file 41598_2018_29177_MOESM1_ESM.pdf]

Mid-infrared plasmonic multispectral filters

Ang Wang[1] and Yaping Dan[1,2]*

[1]State Key Laboratory of Advanced Optical Communication Systems and Networks, University of Michigan-Shanghai Jiao Tong University Joint Institute, Shanghai Jiao Tong University, Shanghai 200240, China
[2]Key Laboratory of Infrared Imaging Materials and Detectors, Shanghai Institute of Technical Physics, Chinese Academy of Sciences, Shanghai 200083, China
*To whom correspondence should be addressed: yaping.dan@sjtu.edu.cn

Supporting information

1. To quantify the errors introduced by the 20% transmission for the other spectral region except for the peak, we shift the filtering function $F(\lambda)$ downside by 20% (keep the negative values at 0) and recalculate the IR spectrum of $CaCO_3$. By comparing the original and recalculated result as shown in Figure S1 we found that the small peaks at around 800 cm^{-1} wavenumber can be distinguished better, which means that the 20% background transmission can reduce the sensitivity of the IR spectrum analyzer based on Au hole arrays filter.

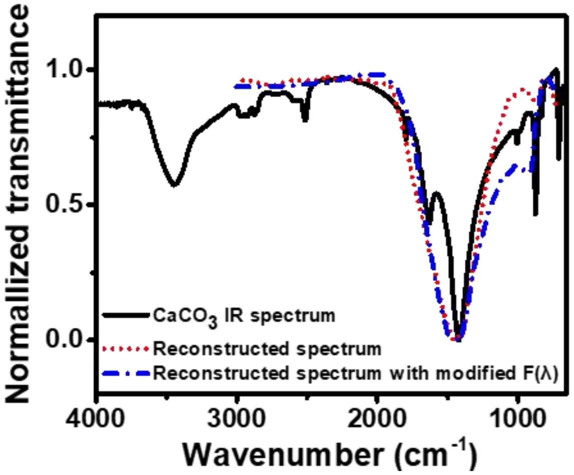

Figure S1: Spectrum of $CaCO_3$ and calculated photocurrent ratio with the original transmission spectrum of each hole array and the modified filtering function.

2. To choose the best ratio of period to diameter from simulation results in Figure 1 (b), we calculated the ratio of maximum transmission to FWHM as shown in the table below. Note that the transmission of the other spectral region does not decay to zero and was deducted from the maximum transmission in this calculation since the filtering performance is mainly determined by the main peak of the filtering spectrum.

Ratio of diameter to period	**0.6**	**0.55**	**0.5**	**0.45**	**0.4**
Maximum transmission	42%	36%	32%	24%	15%
FWHM	1.75	1.38	0.99	0.77	0.5
Ratio	0.24	0.26	0.32	0.31	0.30

From this table we can easily find that the best ratio of period to diameter is 0.5.

3. The microholes are all in shape of circle and arrayed in a hexagonal lattice, so that the transmission spectra stay consistent for different polarizations of the light source. Further simulations with y-polarized and circular polarized light have been performed to prove this. The transmission spectra are almost same with the one in Figure 1 (a) and (b). The simulated transmission spectra and E-field distribution inside the holes at peak position in these two cases are shown in Figure S2, respectively.

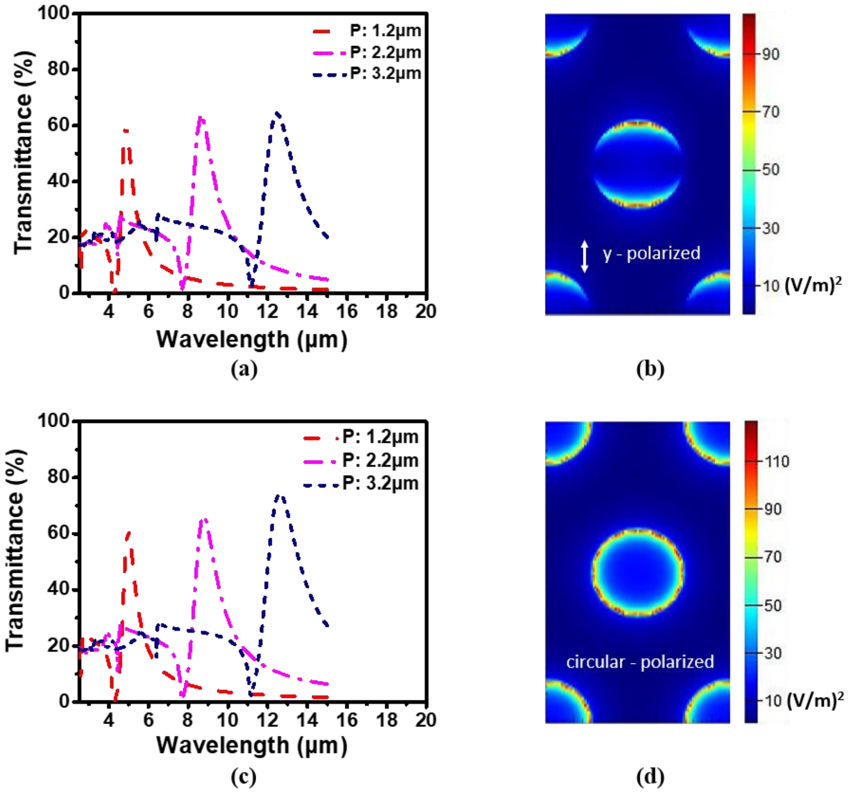

Figure S2: (a) (c) Simulation results of Au MHAs with a fixed ratio 0.5 for aperture diameter to period and (a) y-polarized light source; (c) circular polarized light source; (b) (b) Top view of the simulated E-field distribution inside the holes at peak position with (b) y-polarized light source and (d) circular polarized light source.
